# Evidence of Protein Adsorption in Pegylated Liposomes: Influence of Liposomal Decoration

**DOI:** 10.3390/nano7020037

**Published:** 2017-02-10

**Authors:** Marc Sangrà, Joan Estelrich, Raimon Sabaté, Alba Espargaró, Maria Antònia Busquets

**Affiliations:** 1 Department of Pharmacy, Pharmaceutical Technology and Physical Chemistry, Faculty of Pharmacy and Food Sciences, University of Barcelona, Avda. Joan XXIII, 27-31, 08028 Barcelona, Spain; samglaurung@gmail.com (M.S.); joanestelrich@ub.edu (J.E.); rsabate@ub.edu (R.S.); aespargaro@ub.edu (A.E.); 2 Nanoscience and Nanotechnology Institute (IN2UB), Avda. Joan XXIII, 27-31, 08028 Barcelona, Spain

**Keywords:** magnetoliposomes, liposomes, BSA, protein corona, fluorescence anisotropy, fluorescence quenching

## Abstract

In order to contribute to a better knowledge of the events involved in the formation of the protein corona when nanoparticles (NPs) come in contact with proteins, we report a study about the changes on the physicochemical properties of pristine, PEGylated and Cyclic Arginine-Glycine-Aspartate peptide (RGD)-functionalized large unilamelar liposomes (LUVs) or magnetoliposomes (MLs) upon incubation with Bovine Serum Albumin (BSA). The main phospholipid component of both LUVs and MLs was l-α-phosphatydylcholine (PC) or 1,2-dimyristoyl-*sn*-glycero-3-phosphocholine (DMPC) with 20% of cholesterol. The most obvious indication of the interaction of BSA-nanosystems is given by changes in the hydrodynamic diameter of the particles but other evidence is needed to corroborate the process. Our findings indicate that size modification is a process that is accomplished in few hours and that is strongly dependent not only on the surface decoration but also of the lipid composition of both LUVs and MLs. Fluorescence quenching experiments as well as cryogenic transmission electron microscopy (Cryo-TEM) images assessed these changes and confirmed that although each system has to be studied in a particular way, we can establish three distinctive features that turn into more reactive systems: (a) compositions containing PC compared with their DMPC counterparts; (b) the presence of PEG and/or RGD compared to the pristine counterparts; and (c) the presence of SPIONs: MLs show higher interaction than LUVs of the same lipid composition. Consequently, PEGylation (that is supposed to make stealth NPs) actually fails in preventing complete protein binding.

## 1. Introduction

Liposomes are among the first colloidal soft particles described and could be considered one of the pioneers of the current nanoparticle family. Since their discovery by the end of the 1950s, their application has kept growing for their vast potential in several fields, mainly biomedical [1,2,3,4,5,6]. One of the most interesting features of these biocompatible and biodegradable lipid vesicles is related to their tunable composition that can be designed for a specific purpose and has resulted in a continuous evolution of liposome structure in recent years. Initially, the basic changes to modulate bilayer properties were related to modifications in phospholipid composition—namely polar head charge, saturation, length of the acyl chains, and the introduction of additives such as cholesterol [7,8,9]. However, these changes could not solve one of the main drawbacks that appeared upon liposome administration in vivo which is their rapid recognition and uptake by cells of the mononuclear phagocyte system leading to their quick removal and thus, reduced half life time in the blood stream. This problem was partially overcome by the decoration of the outer leaflet of the bilayer with anti-opsonization agents mostly polymers, especially polyethylene glycol (PEG) generating thus the commonly known as stealth liposomes [10,11,12]. PEG creates a steric barrier between the liposomal surface and the biological medium resulting in a shielding of these nanostructures from immunological recognition and loss of stability. By the PEG approach, surface-surface interactions including the aggregation of liposomes and/or adsorption of plasma proteins are reduced [13] but not totally prevented [14]. More recently, with the main aim of effectively delivering drugs and improving their therapeutic index and reducing their undesired side effects, liposomes have been tailored by attaching particular components to the outer part of the bilayer like peptides [15,16,17] to reach specific targets such as cancer or malaria-infected cells [18,19,20]. Among the peptides, the Arginine-Glycine-Aspartate (RGD) triad has been often selected as nanoparticles scaffold for displaying a strong affinity and selectivity to the α_V_ β_3_ integrin that is overexpressed in malignant tissues. Therefore, RGD attachment to Large Unilamellar Vesicles (LUVs) or Magnetoliposomes (MLs) surface may provide efficient vehicles for therapeutics or contrast agents for the treatment or diagnosis, respectively, of diseases such as thrombosis, osteoporosis, and cancer [21,22].

From all the information mentioned above, it could be concluded that based on the properties of the target, liposomes could be easily and successfully designed for a specific drug delivery purpose or as diagnostic systems. However, studies in vitro and in vivo have shown that, in many occasions, liposomes do not reach the expected location upon their contact with the biological environment as a consequence of the rapid adsorption of proteins, thus immediate formation of a protein shell or corona around the particles is prevented [23,24,25]. Consequently, liposomes with very specific targeting moieties on their surface do not always result in an improved accumulation at the target tissues [26]. It is also known that the characteristics and properties of the corona are strongly dependent on protein source to determine the fate of particle uptake as has been described lately [27,28].

Several surface modifications have been developed in order to modulate or minimize the protein corona. The first one has already been mentioned and consists on the introduction of PEG groups in the lipid bilayer, but the success is not complete because of the residual protein adsorption and the uncertainties in the mechanism of PEG-lipid stabilization. It has also been described that zwitterionic nanoparticles lack a protein corona [29]. Nevertheless, corona formation cannot be completely prevented even with the presence of PEG that reduces protein adsorption without lack of cellular uptake [30].

The determination of the corona and the comprehension of its dependence on the nanoparticle properties has become of capital importance in nanoscience as can be stated by the large amount of published papers in the last few years. In this aspect, Lundqvist et al. [31] have reported that even for a specific material type, the size of the particle, and its surface modification can completely alter the protein corona and probably their biological impact. Corona proteins can physically mask the nanoparticle surface, potentially affecting the therapeutic effect of the molecules bound to the nanoparticle surface [32]. On another hand, it has also been described that cellular uptake is strongly related to the nature of the proteins that constitute protein corona [33]. For instance, it has been found that albumin pre-loading increased the macrophage uptake of PPEylated(polyphosphoester) nanocarriers while it was decreased when these same nanocarriers where loaded with clusterin. Schöttler et al. [34] have found that, in presence of proteins, PEGylated nanocarriers incubated with 10% of fetal bovine serum were not taken up by HeLa cells in contrast with the high uptake observed in absence of a previous incubation. Not only altered plasma protein composition due to different diseases affects the protein corona but also, the composition of the corona varies among healthy individuals [35]. Thus, the protein source is a crucial factor in the formation of the corona.

The protein corona-nanoparticle conjunction has raised the interest of many groups regarding not only liposomes but other organic (nanorods, carbon nanotubes) or inorganic particles (superparamagnetic iron oxide nanoparticles, gold nanobones) [36,37,38,39]. However, even if the protein corona can be understood as a problem in nanoparticle uses for biomedical applications, one can take profit of this apparent drawback and turn what is a problem into a new strategy for developing formulations for a specific purpose [35,40,41,42]. Recently, new molecular targets have been described from identification of the type of proteins that form the corona [43]. In the same trend, fluids enriched with hard corona proteins favored the faster release of the payload compared to those bearing soft coronas [36]. This new approach, attractive in view to the development of personalized medicine, requires a good knowledge of the nature of the proteins that form the corona and of the mechanism of protein-nanoparticle interaction [44,45].

Considering the aforementioned, and due to the insufficient knowledge about the interaction of liposomes with the biological environment—which make difficult any prediction of the biological outcomes—we present a study of the behavior of LUVs and hybrid LUVs containing superparamagnetic iron oxide nanoparticles (SPIONs), named magnetoliposomes (MLs), in presence of bovine serum albumin (BSA). BSA binds to nanoparticles and changes their physicochemical properties. The BSA-nanoparticles complex has enhanced penetration capability in the biological membrane over bare nanoparticles. To analyze the influence of surface composition on the interaction, these systems were decorated with polyethylene glycol (PEG) or the cyclic peptide RGD. Therefore, we report the results obtained with three types of LUVs or MLs: pristine, PEGylated, and RGD functionalized. l-α-phosphatydylcholine (PC) and 1,2-dimyristoyl-*sn*-glycero-3-phosphocholine (DMPC) were the main phospholipids chosen for the synthesis of the lipid vesicles for their differences in the acyl chain. The BSA-LUVs or BSA-MLs interactions were characterized by several methods such as fluorescence, isothermal microcalorimetry (ITC), dynamic light scattering (DSC), transmission electron microscopy (TEM and Cryo-TEM), and sodium dodecyl sulfate polyacrylamide gel electrophoresis (SDS-PAGE). These independent techniques provided similar qualitative results indicating that BSA interactions with LUVs and MLs are strongly dependent on lipid composition. Our findings are in agreement with the reports of several authors that conclude about the difficulty of drawing a general trend and that all the systems need to be studied in a particular way. However, from our studies, we can draw three facts that seem to guide the interactions and increase the magnitude of the binding: (a) compositions containing PC compared with their DMPC counterparts; (b) the presence of PEG and/or RGD compared to the pristine counterparts and; (c) the presence of SPIONs: MLs show higher interaction than LUVs of the same lipid composition.

## 2. Results and Discussion

### 2.1. Physicochemical Characterization of Liposomes and Magnetoliposomes

**Figure 1:**
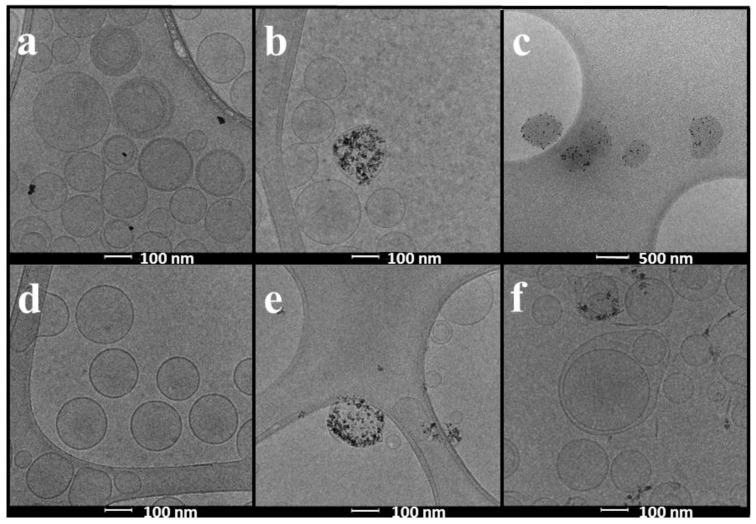
Cryo-TEM images of: (**a**) PC-RGD-LUVs; (**b**) PC-RGD-MLs; (**c**) PC -RGD-MLs incubated 24 h with BSA; (**d**) DMPC-RGD-LUVs; (**e**) DMPC-RGD-MLs; and (**f**) DMPC -RGD-MLs incubated 24 h with BSA. Scale in all the images is 100 nm except for (**c**) which is 500 nm.

The lipid composition, hydrodynamic diameter (*h*_d_), polydispersity index (PDI), and ζ of pristine, PEGylated, and RGD conjugated LUVs or MLs are summarized in Table 1. These measurements were made prior to BSA incubations to set them as control values. All formulations presented low PDI values (<0.200) indicating a mono-modal and narrow size distribution. TEM and Cryo-TEM images showed (see Figure 1) well-dispersed round-shaped vesicles with a size that correlates that of dynamic light scattering (DLS) measurements.

### 2.2. Physicochemical Characterization of BSA Corona-Coated Liposomes and Magnetoliposomes

Serum albumins are the major soluble protein constituents of the circulatory system and are essential for several physiological functions as the transport of a number of compounds. Therefore, the understanding of its interaction with lipid vesicles could contribute to the design of liposomes with a particular composition to improve the delivery of drugs to a specific target. Bovine serum albumin (BSA) has been chosen for the present study due to its structural homology with human serum albumin (HSA).

Protein corona was allowed to form onto vesicles by incubating the LUV or ML formulations with BSA at 37 °C for 2, 4, or 24 h. No significant changes were observed at these incubation times thus confirming the data found in the literature where several authors indicate the formation of the protein corona very quickly after the incubation of other types of nanoparticles with plasma proteins [25].

Isolation of liposomes from loosely-bound and unbound BSA was performed by size exclusion chromatography. Strong centrifugation was excluded to prevent alterations in vesicle-protein interactions.

#### 2.2.1. Changes in Size, Polydispersity Index, and Zeta Potential Corroborate the Interaction of BSA with LUVs and MLs

The formation of the protein corona is usually manifested as an increase in the mean nanoparticle diameter since a layer of protein molecules is deposited onto their surface. In the case of liposomes, the interaction with proteins is more complex as a consequence of their elastic and softer structure which can result in two opposite effects, a reduction due to an osmotically driven shrinkage or an increase in their *h*_d_ [42]. According to this statement, our results indicate a different behavior depending on not only lipid composition but also on the presence or not of SPIONs into the liposomes (Table 2).

The results indicated in the table correspond to increases in the *h*_d_, PDI, and ζ potential after incubation with BSA for a period of 24 h at 37 °C, with respect to the initial values before incubation. Of the two phospholipid compositions studied, it is clear that the samples containing PC are unstable in the presence of BSA. Aggregation and formation of a thick precipitate was visible just after LUVs/MLs-BSA mixing and it was impossible to separate free BSA from BSA-LUVs or MLs complexes by size exclusion chromatography. Contrarily, DMPC LUVs are stable regardless of the surface decoration. The presence of SPIONs in the same lipid composition creates a different behavior. Pristine and PEGylated MLs become slightly larger without PDI significant changes indicating that the samples remain homogeneous, while RGD-DMPC MLs are clearly unstable in the presence of BSA behaving as PC samples. According to the predictions of the dense spherical model [46] the smallest aggregate containing two identical spheres of diameter *h*_d_ results in a particle with size corresponding to 2*h*_d_. Therefore, these little changes in size are not compatible with the formation of a thick corona [47] and could reflect the particle coating with a thin layer of BSA.

ζ potential was not possible to determine in the case of aggregated samples and, in general, becomes more negative than the original samples in agreement with literature reports assessing the presence of BSA in the outer leaflet of the liposome [43].

TEM and Cryo-TEM have been used to visualize the changes associated to the protein corona formation. As an example of the most evident interaction, we show in Figure 1 images of LUVs (left) and MLs (middle) of PC-RGD (top panel) and DMPC-RGD (bottom panel). The pictures on the right panels correspond to MLs incubated 24 h, at 37 °C, with BSA. It can be seen how, after incubation, morphology of the MLs is dramatically altered, especially for the PC composition (500 nm scale in contrast to the other images in which it is of 100 nm).

#### 2.2.2. SDS-PAGE Evidences the Presence of BSA in LUVs and MLs

**Figure 2:**
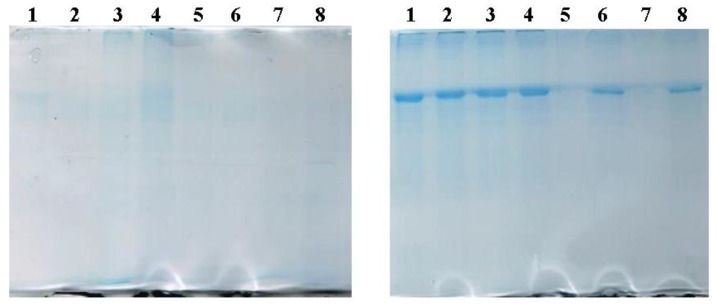
SDS-PAGE of: (**left**) LUVs and (**right**) MLVs after 24 h incubation with BSA at 37 °C. The order of the bands is from left to right: 1: Bare PC; 2: bare PC/Chol; 3: PEGylated-PC; 4: RGD-PC; 5: bare DMPC; 6: bare DMPC/Chol; 7: PEGylated-DMPC; and 8: RGD-DMPC.

BSA associated with the samples after incubation for 24 h was separated by SDS-PAGE and visualized with Coomassie Blue staining (Figure 2). The panel on the left corresponds to the incubation of LUVs while the one on the right refers to the incubation with MLs. SDS-PAGE of MLs show a greater intensity of the BSA band compared to its non-magnetic counterpart, suggesting that the presence of SPIONs on the vesicles also affects the interaction between the nanoparticle and BSA. PC-MLs bands (1–4, right panel) showed higher intensity than DMPC-MLs (5–8, right panel). Among the last ones, it is difficult to understand why the DMPC-MLs band (5) shows less intensity than the DMPC-Chol band (6). It could be attributed to previously-mentioned observation that, in general, DMPC samples are less reactive and the differences observed among DMPC-MLs are not significant. To discard the influence of pure phospholipids in the interaction, bare PL-LUVs or PL-MLVs (PL: PC or DMPC) were also applied to the gel.

#### 2.2.3. Fluorescence Analysis of BSA Binding to LUVs or MLs

##### Fluorescence Quenching

BSA has two intrinsic tryptophan (Trp) residues, one at the surface exposed to the medium and another buried inside the protein [48] that act as a fluorophores. The changes in fluorescence intensity of both Trp residues may be due to a direct quenching or as a consequence of a change on the protein conformation upon BSA-Lipid interaction. The fluorescence quenching refers to any process that decreases the intensity of a fluorophore caused by a variety of molecular interactions with a quencher molecule and depends upon the accessibility of the quencher molecules to the fluorophores.

**Figure 3:**
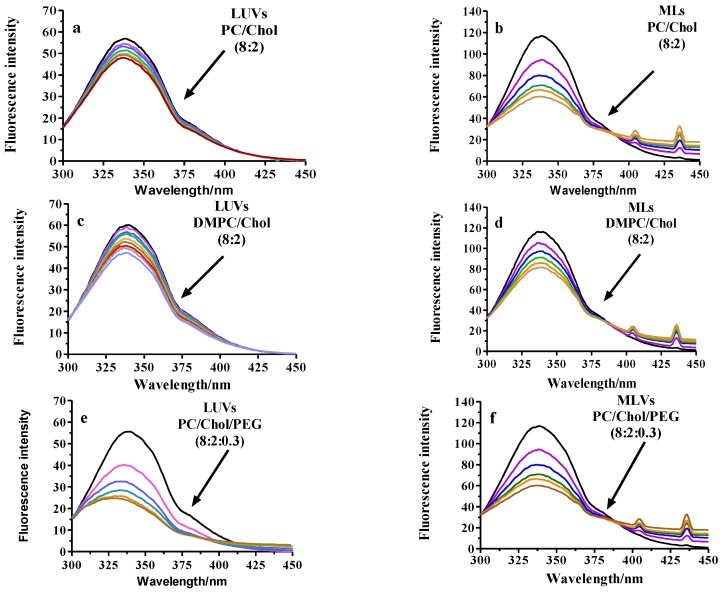

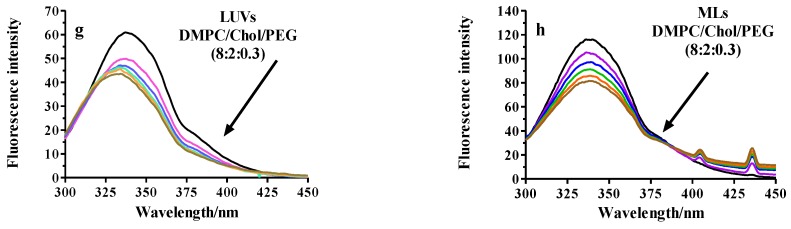
Fluorescence spectra obtained after the incubation of BSA with PC or DMPC LUVs or MLs. (**a**) LUVs PC/Chol (8:2); (**b**) MLs PC/Chol (8:2); (**c**) LUVs DMPC/Chol (8:2); (**d**) MLs DMPC/Chol (8:2); (**e**) LUVs PC/Chol/PEG (8:2:0.3); (**f**) MLVs PC/Chol/PEG (8:2:0.3); (**g**) LUVs DMPC/Chol/PEG (8:2:0.3); (**h**) MLVs DMPC/Chol/PEG (8:2:0.3). The black line corresponds to the spectrum of 5 µM free BSA while the rest refer to the recordings after consecutive additions of 5 µL of 20 mM LUVs or MLs. The arrow indicates the direction of increase in LUVs or MLs concentration ranging from 62 to 370 µM into the reaction cuvette. λ_exc_: 280 nm. The intensity of the emission band at 338 nm corresponding to the tryptophan was used for the calculation of the Stern-Volmer constant (*K*_SV_) [51].

Fluorescence quenching is a useful approach to get insight into the understanding of the interaction of compounds of several sources with body proteins [49]. Thus, fluorescence quenching has been used to measure the binding affinities between the liposomes and BSA. Figure 3, illustrates the influence of the presence of LUVs of different composition on the fluorescence spectra of BSA and Table 3 shows a summary of the results after treatment of the data according to Equations (1) and (2). The fluorescence intensity decreased gradually with the increase in the concentration of LUVs or MLs, implying an interaction between the protein and the lipid vesicles.

We assume that the changes in the BSA spectrum come from their interaction with the lipids and that the quenching constant can be taken as the binding constant of the complex formation [50]. The calculations were based on the changes of the maximum fluorescence emission wavelength (λ_max_) of BSA that was found to be 338 nm. For all the samples, the lipid addition results in a decrease in the fluorescence intensity maximum in a similar magnitude except for the PEGylated samples and, especially, for PEGylated-PC (Figure 3). The decrease occurs just after the first addition that corresponds to a PEGylated-PL/BSA molar ratio of ~12.5/1. This modification of fluorescence intensity might be due to the quenching of the Trp residues present in the BSA molecule. The fluorescence quenching is usually classified in two types, dynamic or collisional and static. Dynamic quenching results from the diffusion of the quencher and the fluorophore in the medium and does not modify the absorption spectra because it only affects the excited state of the fluorophore. Upon the contact, the fluorophore returns to the ground state without emission of a photon. Therefore, a dynamic quencher agent provides a non-radiative route for loss of the excited state energy. On another hand, static quenching is a consequence of the formation of a dark complex (non-fluorescent) in the ground state between the quencher molecules and the fluorophore that is assessed by a change in the absorption spectra.

Both, dynamic and static quenching require molecular contact between the fluorophore and the quencher and can be distinguished in the plot *F*_o_/*F* vs. quencher concentration. Dynamic quenching is characterized by a straight line while static appears as a positive deviation. In our case, all the samples bended towards the *x*-axis, indicative of a negative deviation and consequently, static quenching was discarded. This kind of deviation is common in systems having accessible and inaccessible fluorophores, as it could be the presence of more than one Trp. That is the case of BSA with two Trp residues: Trp-134 located on the surface of the molecule, and Trp-212 buried in a hydrophobic binding pocket. However, the deviation is probably a consequence of LUV or ML aggregation due to the turbidity observed in the samples at high lipid concentrations.

Particularly interesting are the results obtained when the titration is made with PEGylated-PC because the decrease in fluorescence intensity is accompanied by a blue shift. This change reaches 12 nm when the molar ratio PEGylated-PC/BSA is ~300/1. In that case, apart from the quenching phenomena, spectral shifting is associated to Trp exposure to changes in the polarity as a consequence of the interactions with its environment. Thus, polarity of the chromophores of the BSA (two Trp residues present in each BSA molecule) decreased and the hydrophobicity increased. The quenching mechanism can be unravelled from the Stern-Volmer equation (Equation (1)) that allows the calculation of *K*_SV_. Although strong ligand-protein complexes are characterized by lipid-BSA association constants (*K*_SV_) with values ranging from 10^5^ to 10^8^ M^−1^, some authors have reported interactions with lower *K*_SV_ values consistent with our results [51]. According to the Hill model [52], *K*_SV_ can be considered to be reciprocal to the dissociation constant *K*_D_ (≈1/*K*_SV_) for certain conditions though *K*_D_ refers to equilibrium and 1/*K*_SV_ to non-equilibrium conditions. We have not considered this approach because of the evidence of only collisional quenching and the lack of trustable results when calculating *K*_D_ from *K*_SV_ [53]. In addition, Fleischer and Payne [54] pointed out that data obtained with physicochemical experiments were significantly different from those found with other methods, consequently suggesting caution in assuming that *K*_D_ can be deduced from *K*_SV_. In agreement with previous results, ML samples showed more significant changes in BSA spectra and consequently, *K*_SV_ is higher. Remarkable and unexpected was the high value of the Stern-Volmer constant for PEGylated-PC reflecting a higher BSA binding compared to the other lipid compositions. Consequently, PEGylation (that is supposed to make NPs stealth) actually fails in preventing complete protein binding. On another hand, fluorescence spectra of BSA remained unchanged upon titration of the cuvette with SPIONs. This indicates that BSA binding to the nanoparticles is only related to the properties of the outer leaflet of the bilayer determined by the lipid composition and the attached peptide.

##### Fluorescence Anisotropy

The purpose of the anisotropy measurements was to analyze the structural changes on the lipid membrane during BSA binding to LUVs or MLs. Thus, the dynamics of lipids in LUV or ML membranes in the presence of BSA was determined by measuring the degree of depolarization of the fluorescence (Equation (2)) emitted from the hydrophobic probe TMA-DPH, which is anchored at the water/lipid interface due to its charged trimethylammonium group. *r* values are high at temperatures below the main transition of the lipid, from gel to liquid crystalline state due to the highly-restricted rotation for the probe. Experiments were only performed with the samples containing DMPC because of its well-defined *T*_m_ at 23 °C in contrast with PC that has a *T*_m_ below 0. *r* remained unchanged after BSA addition to the cuvette that contained DMPC-LUVs or DMPC-MLs, regardless of the surface composition. Considering that the working temperature was above *T*_m_, the lack of changes in *r* could be attributed to an increase in surface rigidity due to the presence of BSA around the LVs or MLs.

#### 2.2.4. Thermotropic Behavior of LUVs and MLs in Presence of BSA

The calorimetric experiments were only performed with the samples containing DMPC as the main phospholipid for having well-defined thermotropic properties in contrast to those of PC.

##### Differential Scanning Calorimetry (DSC)

In lipid bilayers, lipid phase behavior is sensitive to the presence of exogenous compounds on surfaces and this effect was considered as a tool to study the interaction of LUVs and MLs with BSA. As far as the controls without BSA are concerned, DMPC exhibited the characteristic main transition at ~24.4 °C, the addition of a 20% cholesterol resulted in the appearance of a shoulder at a higher temperature indicative of a broadening of the melting region while the main transition disappeared upon the addition of a 3% PEG. Consequently, RGD presence gave the same profile as the DMPC/CHOL/DSPC-PEG mixture. For that reason, changes due to BSA presence in the media was only evaluated with DMPC or DMPC/Chol samples. Significant differences were observed between LUVs and MLs. BSA did not change either the shape or the energy of DMPC main transition in LUVs. However, this transition completely disappeared in MLs and could be attributed to the presence of the encapsulated SPIONs.

##### Isothermal Titration Calorimetry (ITC)

The binding properties of the LUVs and MLs with BSA were consecutively studied using ITC. The total heat produced endothermally was plotted while injecting BSA in LUVs or MLs at 37 °C (Figure not shown). The molar ratios PL/BSA ranged from 0.28/1 in the first addition to 7.1/1 after the last (30th), similar to those studied in the fluorescence section. Results did not show any significant binding and no thermodynamic parameters could be obtained as shown for the lack of differences between the heats for the LUVs or MLs and the dilution heats of BSA. Thus, the binding of the LUVs or MLs seem to be weak as observed with fluorescence experiments.

## 3. Methods

### 3.1. Materials

1,2-dimyristoyl-*sn*-glycero-3-phosphocholine (DMPC), 1,2-distearoyl-*sn*-glycero-3-phosphoethanolamine-*N*-[methoxy(polyethylene glycol)-2000] (ammonium salt) (DSPE-mPEG-2000), and 1,2-distearoyl-*sn*-glycero-3-phosphoethanolamine-*N*-[maleimide (polyethylene glycol)-2000] (ammonium salt) (DSPE-Mal-PEG-2000) were obtained from Avanti Polar Lipids (Alabaster, AL, USA) and cholesterol (CHOL) from Sigma (Barcelona, Spain). l-α-phosphatydylcholine (PC) was procured from Lipoid (Ludwigshafen, Germany). Cyclic RGD-(d-Phe)-C (with an amide bond) peptide was synthesized by CASLO Laboratory ApS (Kongens Lyngby, Denmark). Iron (II) chloride tetrahydrate (FeCl_2_·4H_2_O) and iron (III) chloride hexahydrate (FeCl_3_·6H_2_O) were purchased from Sigma Aldrich (St. Louis, MO, USA). Polyethylene glycol (PEG) of 6000 Da average molecular weight was obtained from VWR International (Barcelona, Spain). Ammonium hydroxide (NH_4_OH, 25%) was provided by Panreac (Barcelona, Spain). Deionized Millipore Milli-Q water was used in all experiments. Bovine Serum Albumin (BSA) was purchased from Sigma Aldrich (St. Louis, MO, USA). The fluorescent probe 1-(4-trimethylammoniumphenyl)-6-phenyl-1,3,5-hexatriene *p*-toluenesulfonate (TMA-DPH), was from Molecular Probes, Inc (Eugene, OR, USA). A strong neodymium-iron-boron (Nd_2_Fe_12_B) magnet (1.2 T) was obtained from Halde GAC (Barcelona, Spain). All other chemicals were from Sigma (St. Louis, MO, USA), were of analytical grade, and were used without purification.

### 3.2. Methods

#### 3.2.1. Preparation and Characterization of Liposomes and Magnetoliposomes

PL and mixtures of PL/CHOL (8:2 molar ratio), PL/CHOL/DSPE-PEG (8:2:0.3 molar ratio) or PL/CHOL/DSPE-PEG-Maleimide/RGD (8:2:0.3:0.03 molar ratio), being PL: PC or DMPC, were prepared by dissolving appropriate amounts of lipid in chloroform/methanol (2:1 *v*/*v*). After solvent evaporation under reduced pressure, the resulting lipid films were hydrated with ultrapure water and after soft sonication in a bath sonicator, LUVs were prepared by the extrusion of the preparations 10 times through 200 nm pore-size poly-carbonate filters (Nucleopore) in a low pressure extruder (Lipex, Biomembranes, Vancouver, BC, Canada). The phospholipid content was determined by the Steward–Marshall method [55]. The calibration curve was performed with the same different lipid mixtures in chloroform that the used in the study. Absorbance was measured in a Shimadzu UV-2401 PC UV-vis spectrophotometer (Shimadzu, Tokyo, Japan). For the preparation of MLs, a similar procedure was followed but the lipid film was hydrated with a ferrofluid (FF) solution, a suspension of SPIONs instead of water. Non encapsulated FF was separated from the loaded into liposomes by gel exclusion chromatography with Sepharose 4B (Pharmacia Fine Chemicals, Uppsala, Sweden). FF was prepared according to the method described by García-Jimeno et al. [56]. Iron content was determined spectrophotometrically following the method of Kiwada [57].

LUVs or MLs size before and after incubation with BSA was measured at different times from their preparation depending on the assay. 2% (*w*/*v*, ~0.31 mM) BSA and 25 mM LUVs or MLs were mixed at a ratio of 1:1 (*v*/*v*), resulting in a final PL/BSA molar ratio of ~83/1. The hydrodynamic diameter (*h*_d_) and the corresponding polydispersity index (PDI) were determined by dynamic light scattering at a fixed scattering angle of 90° with a Zetasizer Nano (Malvern, UK) at 25 °C and 37 °C. LUVs or MLs from the stock solution were dispersed in water to obtain approximately 0.1 g·L^−1^ solid content.

Geometry of LPs and MLs was observed by transmission electron microscopy (TEM) and cryo-TEM. For TEM observations, a Jeol 1010 microscope (Jeol, Tokyo, Japan) operating at 80,000 kV was used. Samples were prepared by placing a drop of MLs onto a 400-mesh copper grid coated with carbon, and after staining with uranyl acetate they were allowed to dry in the air before being placed into the microscope. Images were recorded with a Megaview camera. Acquisition was accomplished with the Soft-Imaging software (SIS, Schwentinental, Germany). For cryo-TEM observations, grids were transferred to a Tecnai F20 (FEI, Eindhoven, The Netherlands) using a cryoholder (Gatan, Warrendale, PA, USA). Images were taken at 200 kV, at a temperature ranging from −175 to −170 °C and using low-dose imaging conditions with a 4096 × 4096 pixel CCD Eagle camera (FEI, Eindhoven, The Netherlands).

The zeta potential of the dispersion was measured diluted in 10^−3^ M potassium chloride solution with a Zeta Sizer Nano Series (Malvern Instruments, Worcestershire, UK) at 20 °C.

#### 3.2.2. Isothermal Titration Calorimetry (ITC)

ITC measurements were performed using a MicroCal VP-ITC (Northampton, MA, USA) with an effective cell volume of 1.4199 mL and feedback mode: high. The experimental temperature was kept constant at 37 °C. The number and injected volume of the titration steps were the same for all measurements (30 × 10 μL). The initial delay was set to 60 s.

The working cell was filled with a liposome suspension (containing 0.01 mM lipid) in water and the reference cell with liposome-free water. Ten-microliter aliquots of 0.0625 mM BSA were injected stepwise into the working cell at 340 s intervals.

The generated ITC data were collected automatically by the Windows-based Origin Software also supplied by MicroCal (one set of sites binding model) which uses a nonlinear least-squares algorithm (minimization of *X*^2^). To fit the heat flow per injection into an equilibrium binding equation, the software uses titrant and sample concentrations. It provides best fit values of the stoichiometry (*n*), involved enthalpy (Δ*H*_b(ITC)_), and binding constant (*K*_b(ITC)_) at working conditions. Additionally, the same amount of BSA was titrated into pure water to determine the heat of dilution for reference. The integrated reference heats were then subtracted from the integrated heats of the adsorption experiments. All titrations were measured in triplicates. The corresponding reference blank experiments were also performed, namely titration of the liposome suspension with water and titration of water with BSA solution.

#### 3.2.3. Differential Scanning Calorimetry (DSC)

Differential scanning calorimetry was carried out by means of a Metller-Toledo DSC-822e calorimeter. Experimental conditions were as follows: aluminum crucibles of 40 μL volume; atmosphere of dry nitrogen with 50 mL/min flow rate; heating rates of 1–40 °C/min. The concentration of lipids was 20 mM. Data acquisitions measured at least three scans for lipid DMPC were collected between 5 °C and 45 °C at 10 °C/h. Buffer subtraction and baseline correction were performed using Microcal Origin software (Microcal Inc. Worcestershire, UK). The calorimeter was calibrated with indium of 99.99% purity. Lipid and BSA were mixed at a molar ratio of 83/1.

#### 3.2.4. SDS Polyacrylamide Gel Electrophoresis (SDS-PAGE)

30 μL of the BSA incubated liposomes were added to 10 μL of 4× loading buffer [180 mM Tris-HCl pH 6.8, 40% glycerol, 0.05% bromophenol blue, 10% sodium dodecyl sulfate (SDS) and 20% β-mercaptoethanol] and the mixture was heated at 95 °C for 10 min. After centrifugation of the samples ~3 min at 8000 rpm (Eppendord centrifuge) they were resolved on 12% SDS-PAGE gels, stained with Coomassie Brilliant Blue and scanned at high resolution.

#### 3.2.5. Fluorescence Spectroscopy

Fluorescence experiments were performed in an Aminco Bowman AB2 (Microbeam, SA, Barcelona. Spain) spectrofluorimeter adapting the procedure described by Charbonneau and Tajmir-Riahi [58]. A 5 μM BSA solution (in double distilled water) was titrated with different volumes of liposomes (initial concentration 20 mM). The fluorescence spectra were recorded in a range of 300 to 450 nm at an excitation wavelength (λ_ex_) of 280 nm (slit-widths: 4 nm). Then, the quenching of BSA in the presence of LUVs was calculated from the changes on the tryptophan (Trp) fluorescence of the protein at 338 nm (BSA maximum fluorescence emission intensity) with the Stern-Volmer equation (Equation (1))

(1) $$\frac{F_{o}}{F}=1+K_{\mathrm{sv}}\;\left[Q\right]$$

where *F*_o_ and *F* are the steady state fluorescence intensities in absence and presence of liposomes (quencher in our case), respectively, *K*_sv_ is the Stern-Volmer quenching constant.

Corrections for dilution were done by repeating the experiments after adding the same volumes of water to the cuvette containing BSA.

#### 3.2.6. Fluorescence Anisotropy

Steady-state anisotropy measurements were carried out with the same spectrofluorimeter mentioned above using L-format fluorescence polarizers. The dynamics of LUVs in presence of BSA was determined by measuring the degree of depolarization of fluorescence emitted from the probe TMA-DPH. The excitation (λ_ex_) and emission (λ_em_) wavelengths were 365 nm and 425 nm, respectively (slit-widths: 4 nm). DMPC LUVs or MLs were incubated at 40 °C (above *T*_m_ of the lipid) for at least 1 h before use to allow the probe to be incorporated. The initial concentration of lipid in the cuvette was 150 μM, which corresponds to a ratio lipid/probe of 700/1. BSA was added from a 5 µM stock solution in sequential additions of 5 µL. Anisotropy values were corrected from dilution. Anisotropy (*r*) measurements were done at 25 °C and 37 °C with the same equipment described before and was calculated automatically by the software provided with the instrument, according to Equation (2):

(2) $$r=\frac{I_{Vv}-GI_{Vh}}{I_{Vv}+2GI_{Vh}}$$

where *I_Vv_* and *I_Vh_* are intensities of the emitted polarized light with the emission polarizer parallel (*Vv*) or perpendicular (*Vh*), respectively, to the excitation polarizer and, *G* is the instrument sensitivity ratio towards vertically and horizontally polarized light [59].

Control experiments were performed with BSA alone to check the lack of interference between the emissions of the fluorophores of the protein (Trp) and the probe (TMA-DPH).

## 4. Conclusions

The present study compares the behavior of LUVs and MLs differing on surface properties in presence of BSA by several techniques that take into consideration different experimental conditions such as lipid/BSA molar ratios or physical chemistry properties. The rationale of surface modification with cyclic RGD with enhanced stability over the lineal form was aimed to functionalize MLs and LUVs for nanoparticle tumor targeting to integrins rich in RGD receptors that are over expressed in cancer cells. On another hand, the encapsulation of SPIONs was intended for their use as diagnostic systems. We have shown that the characteristics of liposomes can change upon exposure to BSA, which is strongly dependent on the lipid composition. The presence of the unsaturated PC as the main component of the bilayer makes the systems more reactive in contrast to the saturated DMPC. Moreover, further functionalization of the liposomes via PEGylation and/or attachment of RGD shows an enhancement of the reactivity between liposomes and BSA. This effect may be caused by a different type of interaction occurring. In BSA-Pristine systems, interaction is mainly hydrophobic while for BSA-PEGylated systems interactions is basically electrostatic. Similarly, the incorporation of SPIONs into the nanosystems results in more reactive systems. One general problem for unravelling the doubts remaining in protein-nanosystem interaction is that, although numerous studies exist, many of them are hard to compare, as there is a lack of quantitative parameters which could be used as metrics for direct comparison. In our case, size and *K*_sv_ were the only parameters rendering useful data to distinguish nanosystem-BSA interaction. Qualitative binding was observed with SDS-PAGE that also confirmed the presence of larger amounts of BSA in PC LUVs or MLVs than in DMPC systems. In any case, neither the presence of PEG nor RGD reduce the interaction probably as a consequence of the electrostatic interactions present in these nanoparticles. Therefore, even though the results reported indicate the need to study all the nanosystems in a particular manner, there are three general trends that spread among all tests performed. The presence of SPIONs enhances reactivity between BSA and liposomes, as well as the compositions containing PC compared with their DMPC counterparts, and also the presence of PEG and/or RGD compared to the pristine counterparts. However, these effects need to be studied further in order to obtain more quantitative data and better understand these behaviors. Also the influence of SPIONs in the results is an item to be further investigated.

## Figures and Tables

**Table 1:** Physicochemical characterization of LUVs and MLs of different composition before incubation with BSA: *h*_d_: hydrodynamic diameter; PDI: polydispersity index and ζ: zeta potential. PC: l-α-phosphatydylcholine; DMPC: 1,2-dimyristoyl-*sn*-glycero-3-phosphocholine; PEG: 1,2-distearoyl-*sn*-glycero-3-phosphoethanolamine-*N*-[methoxy(polyethylene glycol)-2000] (ammonium salt); PEG*: 1,2-distearoyl-*sn*-glycero-3-phosphoethanolamine-*N*-[maleimide (polyethylene glycol)-2000] (ammonium salt) (DSPE-Mal-PEG-2000); CHOL: cholesterol, and RGD: Cyclic RGD-(d-Phe)-C. Molar ratios into brackets.

Lipid Composition	*h*_d_/nm		PDI		ζ/mV	
	LUVs	MLs	LUVs	MLs	LUVs	MLs
PC/CHOL (8:2)	170.4 ± 1.0	178.5 ± 6.4	0.090 ± 0.041	0.136 ± 0.048	−1.85 ± 0.85	−7.5 ± 0.43
PC/CHOL/PEG (8:2:0.3)	161.8 ± 3.7	114.0 ± 7.6	0.119 ± 0.032	0.129 ± 0.05	−16.6 ± 0.20	−18.9 ± 4.23
PC/CHOL/PEG*/RGD (8:2:0.3:0.03)	136.2 ± 1.5	175.1 ± 1.4	0.146 ± 0.011	0.200 ± 0.01	−13.0 ± 0.90	−27.0 ± 0.35
DMPC/CHOL (8:2)	180.0 ± 3.6	191.5 ± 6.9	0.122 ± 0.018	0.200 ± 0.02	−4.66 ± 0.92	−1.23 ± 0.96
DMPC/CHOL/PEG (8:2:0.3)	154.9 ± 4.5	168.7 ± 6.6	0.163 ± 0.024	0.139 ± 0.06	−14.8 ± 0.14	−15.7 ± 0.40
DMPC/CHOL/PEG*/RGD (8:2:0.3:0.03)	183.1 ± 4.2	198.0 ± 5.2	0.149 ± 0.023	0.135 ± 0.02	−26.3 ± 0.95	−22.5 ± 0.83

**Table 2:** Changes in size, polydispersity index (PDI) and ζ potential of LUVs or MLs upon incubation with 1 mg·mL^−1^ (ca. 16 μM) BSA for 24 h. The values are expressed as increases calculated by subtracting the initial value to the value after incubation. Pristine-PC or DMPC: PC/CHOL (8:2) or DMPC/CHOL (8:2); PEGylated PC or DMPC: PC/HOL/PEG (8:2:0.3) or DMPC/CHOL/PEG (8:2:0.3) and RGD-PC or DMPC: PC/CHOL/PEG*/RGD (8:2:0.3:0.03) or DMPCPC/CHOL/PEG*/RGD (8:2:0.3:0.03).

Lipid Composition	LUVs			MLs		
	Δ Size/nm	Δ PDI	Δ ζ/mV	Δ Size/nm	Δ PDI	Δ ζ/mV
Pristine-PC	Aggregated	>1		Aggregated	>1	
PEGylated-PC	Aggregated	>1		Aggregated	>1	
RGD-PC	Aggregated	>1		Aggregated	>1	
Pristine-DMPC	~0	0.017	−25.25 ± 16.53	4.50 ± 2.6	0.070	−4.24 ± 4.35
PEGylated-DMPC	~0	0.014	−0.73 ± 1.00	24.0 ± 5.2	0.085	−16.93 ± 3.59
RGD-DMPC	~0	0.014	−25.25 ± 2.05	Aggregated	>1	

**Table 3:** *K*_SV_ calculated from the fluorescence spectra of BSA upon increased additions of LUVs or MLs of different composition.

Lipid Composition	LUVs		MLs	
	*K*_sv_/L·mol^−1^	*r*	*K*_sv_*/*L·mol^−1^	*r*
Pristine-PC	345 ± 6	0.994	2465 ± 30	0.996
PEGylated-PC	4460 ± 22	0.997	1908 ± 27	0.997
RGD-PC	963 ± 15	0.995	2151 ± 13	0.996
Pristine-DMPC	500 ± 12	0.992	1237 ± 20	0.999
PEGylated-DMPC	840 ± 10	0.996	1685 ± 35	0.997
RGD-DMPC	1300 ± 21	0.994	1739 ± 24	0.999

## Abbreviations

BSA	Bovine Serum Albumin
DMPC	1,2-dimyristoyl-*sn*-glycero-3-phosphocholine
CHOL	Cholesterol
FF	Ferrofluid
*h*_d_	Hydrodynamic diameter
LUVs	Large unilamellar vesicles
MLs	Magnetoliposomes
NPs	Nanoparticles
PC	l-α-phosphatydylcholine
PDI	Polydispersity Index
PEG	1,2-distearoyl-*sn*-glycero-3-phosphoethanolamine-*N*-[methoxy(polyethylene glycol)-2000] (ammonium salt)
PEG*	1,2-distearoyl-*sn*-glycero-3-phosphoethanolamine-*N*-[maleimide (polyethylene glycol)-2000] (ammonium salt) (DSPE-Mal-PEG-2000)
PL	Phospholipid
RGD_c_	Cyclic arginine-glycine-aspartate peptide
SDS-PAGE	Sodium dodecyl sulfate polyacrylamide gel electrophoresis
Cryo-TEM	Cryogenic transmission electron microscopy
